# Accelerating Research With Technology: Rapid Recruitment for a Large-Scale Web-Based Sleep Study

**DOI:** 10.2196/10974

**Published:** 2019-01-21

**Authors:** Sean Deering, Madeline M Grade, Jaspreet K Uppal, Luca Foschini, Jessie L Juusola, Adam M Amdur, Carl J Stepnowsky

**Affiliations:** 1 Veterans Affairs San Diego Healthcare System Health Services Research & Development Unit La Jolla, CA United States; 2 American Sleep Apnea Association Washington, DC United States; 3 Evidation Health San Mateo, CA United States; 4 Stanford University School of Medicine Stanford, CA United States; 5 Department of Medicine University of California at San Diego La Jolla, CA United States

**Keywords:** connected health, engagement, health, mHealth, mobile health, mobile phone, recruitment, sleep, sleep quality, wearables

## Abstract

**Background:**

Participant recruitment can be a significant bottleneck in carrying out research studies. Connected health and mobile health platforms allow for the development of Web-based studies that can offer improvement in this domain. Sleep is of vital importance to the mental and physical health of all individuals, yet is understudied on a large scale or beyond the focus of sleep disorders. For this reason and owing to the availability of digital sleep tracking tools, sleep is well suited to being studied in a Web-based environment.

**Objective:**

The aim of this study was to investigate a method for speeding up the recruitment process and maximizing participant engagement using a novel approach, the Achievement Studies platform (Evidation Health, Inc, San Mateo, CA, USA), while carrying out a study that examined the relationship between participant sleep and daytime function.

**Methods:**

Participants could access the Web-based study platform at any time from any computer or Web-enabled device to complete study procedures and track study progress. Achievement community members were invited to the study and assessed for eligibility. Eligible participants completed an electronic informed consent process to enroll in the study and were subsequently invited to complete an electronic baseline questionnaire. Then, they were asked to connect a wearable device account through their study dashboard, which shared their device data with the research team. The data were used to provide objective sleep and activity metrics for the study. Participants who completed the baseline questionnaires were subsequently sent a daily single-item Sleepiness Checker activity for 7 consecutive days at baseline and every 3 months thereafter for 1 year.

**Results:**

Overall, 1156 participants enrolled in the study within a 5-day recruitment window. In the 1st hour, the enrollment rate was 6.6 participants per minute (394 per hour). In the first 24 hours, the enrollment rate was 0.8 participants per minute (47 participants per hour). Overall, 1132 participants completed the baseline questionnaires (1132/1156, 97.9%) and 1047 participants completed the initial Sleepiness Checker activity (1047/1156, 90.6%). Furthermore, 1000 participants provided activity-specific wearable data (1000/1156, 86.5%) and 982 provided sleep-specific wearable data (982/1156, 84.9%).

**Conclusions:**

The Achievement Studies platform allowed for rapid recruitment and high study engagement (survey completion and device data sharing). This approach to carrying out research appears promising. However, conducting research in this way requires that participants have internet access and own and use a wearable device. As such, our sample may not be representative of the general population.

## Introduction

Participant recruitment can be a significant rate-limiting step to the implementation of research studies [1]. A study found that less than one-third of publicly funded multicenter trials met recruitment targets within the planned timeframe [2]. Another study found that 40% of studies underenrolled participants, with 11% failing to enroll a single participant [3]. Study recruitment can take several months and consume up to 30% of study timelines, which can delay scientific progress and incur substantial additional costs. Another related study found the estimated cost of recruiting and retaining participants for clinical trials alone to be US $2.3 billion annually [4].

The study recruitment process is often underfunded and underresourced. Internet-based technologies have the potential to offer improvement in this domain [5] or, at least, supplement existing approaches. Lessons are being learned from Web-based trials where these processes can be shifted to a less resource-intensive Web-based connected platform. This allows for carrying out faster, cheaper, and more demographically and geographically diverse medical research [4,6,7].

Connected research platforms allow research to be brought out of the traditional lab setting and directly to participants through mobile devices and computers. This approach is part of the larger emerging trend of connected health [8] and mobile health (mHealth) [9]. These platforms may provide a way for participants to be accessed through their mobile devices, through research apps that can be used to monitor health in near real time. Examples of underlying platforms and frameworks that these mobile research apps have been developed based upon include Open mHealth [10], Apple ResearchKit [11], and Android ResearchStack [12]. Apps utilizing these platforms allow survey questionnaires and assessments to be administered to participants quickly and easily. The apps also make it possible to sample consumer wearable data over long periods of time, garnering a great deal of useful data in domains ranging from activity to sleep. The Achievement Studies platform (Evidation Health, Inc, San Mateo, CA, USA) is an example of one such connected platform that is Web-based and, therefore, allows access to participants who are not mobile-enabled or prefer nonmobile Web access; in other words, the platform enables users of any connected device (phone, tablet, laptop, or desktop) to participate in research. This platform has been used to run a wide variety of digital studies [13-15].

There is emerging evidence in support of the myriad ways that these apps and platforms are improving certain aspects of the research process. They have significantly increased the speed and ease of recruiting participants for certain kinds of research studies [13-18], made studies accessible to a larger audience [19], and evidence from prior studies suggests that these app-based interventions promote positive health outcomes in patients with a variety of chronic conditions [15,20-23]. As such, they appear promising for future research studies, but their long-term value remains to be seen.

The motivation for this study was to take advantage of the technological and methodological advancements in the field of connected health or mHealth and apply them to a large-scale research study on sleep. Sleep is an essential biological function of vital importance for many aspects of physical and mental health [24]. The Centers for Disease Control have termed insufficient sleep “a public health epidemic” [25], yet large-scale studies of sleep patterns, quality, and associated characteristics that are substantiated by objective data collection are lacking. Given the importance of sleep health for individuals across demographics and health statuses, the population of interest is maximal. Utilizing a novel platform, the aim of the study was to gain further insights into the relationship between participant sleep and daytime functioning while simultaneously speeding up the recruitment process and maximizing data completeness. The Achievement SleepHealth study was adapted from the SleepHealth Mobile App Study, a mobile research study built on the Apple ResearchKit framework [26], and is considered a substudy of the larger study. The SleepHealth Mobile App Study was limited to iPhone users, whereas the Achievement Study is a Web application-based study that can be accessed by any Web-enabled computer or mobile device.

## Methods

### Study Design and Overview

The Achievement SleepHealth study was implemented using a Web-based platform to screen, consent, and enroll eligible individuals from across the United States to participate in this 1-year long observational study (Achievement Studies, Evidation Health Inc). Participants could access the Web-based study platform at any time from any Web-enabled device to track progress and complete various study-required procedures. While all participants took part in the study remotely, research staff were available by phone or email to answer questions and otherwise provide support at any time during and after the study period.

### Participants and Enrollment

Prospective participants were recruited over a 5-day period using a Web-based strategy within the Achievement community. Members of Achievement can connect their activity trackers and fitness and health apps to the program; as members log activities and use their activity trackers, they accumulate points that are redeemable for monetary rewards. Members can also receive targeted offers to participate in various research opportunities and studies. Members with relevant connected activity trackers were invited through email to participate in the Achievement SleepHealth study. Participants were prompted to visit a website to learn more about the study and verify their eligibility by answering a few Web-based screening questions. If eligible, they completed an electronic informed consent (eConsent) process to enroll in the study. The eConsent document specified that the only data that would be included in the study dataset from their personal device would be from smartphone apps that they specifically permissioned as part of study procedures (eg, Fitbit data). They then went on to complete baseline questionnaires and were invited to connect a wearable activity tracker account if they had not already done so. Participants who completed the baseline questionnaires were subsequently sent a single-item Sleepiness Checker on a daily basis for 7 consecutive days.

### Follow-Up

After the initial study procedures described above, participants were asked to track daily alertness levels for 7 consecutive days every 3 months and track sleep and activity with their wearable devices throughout the 1-year study period.

### Measurements

Study questionnaires and the Sleepiness Checker activity from the SleepHealth Mobile App Study were replicated on the Achievement Studies platform. Most questions were multiple-choice type, with a small number of numeric entry questions (eg, participant age, weight, caffeine, and alcohol consumption).

#### Baseline Questionnaires

Six one-time questionnaires were administered to participants sequentially upon enrollment in the Achievement SleepHealth study. The questionnaires were named as follows: About Me, My Health, My Family, Research Interest, Sleep Habits, and Sleep Assessment. The About Me survey was designed to obtain general background information about study participants (eg, gender, age, weight, socioeconomic status, etc). The My Family questionnaire was composed of questions specific to participant households and contained questions such as primary language spoken at home, household size, and the number of minors living at home. The My Health survey included questions related to participants’ physical health. It focused on participants’ beliefs about the likelihood of developing specific health conditions, and how these health conditions and sleep might be related to one another. The Research Interest Questionnaire comprised a series of questions that were intended to gauge the extent of participants’ previous research exposure, as well as their interest in future research. The final 2 questionnaires were sleep-specific. Sleep Habits focused on questions involving participant sleep routines (average time in minutes taken to fall asleep, the number of naps taken during the day, whether a participant was a morning or evening person), whereas the Sleep Assessment contained questions that were specific to sleep quality and included potential problems arising during sleep, as well as symptoms that could potentially be related to various sleep disorders. A common theme of the surveys was that participants were asked questions about their levels of daytime activity, lifestyle, and other factors that could influence their sleep duration and sleep quality.

**Figure 1:**
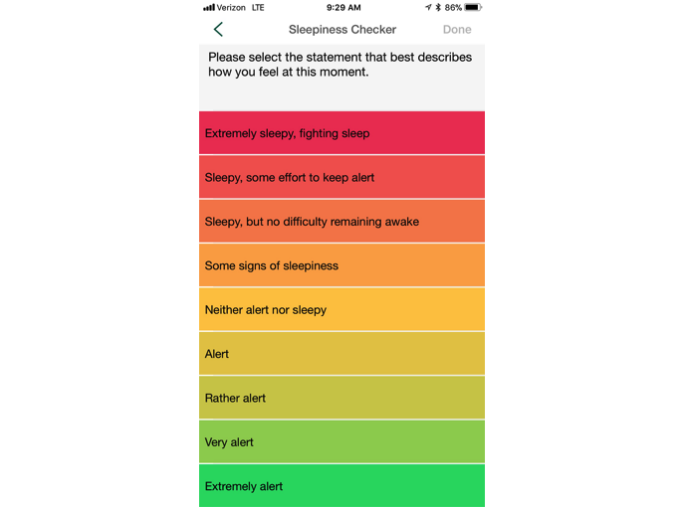
Sleepiness Checker, SleepHealth app.

#### Other Study Measures

##### Sleepiness Checker

Each time the Sleepiness Checker activity was administered to participants (both immediately following the initial questionnaire set and then every 3 months for the 1-year study), it was administered daily for a total of 7 days, sent at the same time of day that the baseline questionnaires were completed. It was adapted from the Karolinska Sleepiness Scale [27], a single-item Likert-type scale anchored by 1 (extremely alert) and 9 (very sleepy, fighting sleep). Figure 1 shows the screenshot of the full range of possible responses.

##### Sleep- and Activity-Specific Data

As the vast majority of participants used the Fitbit wearable, it was considered the study’s primary source of sleep and activity data.

### Data Analysis

This paper focuses on baseline characteristics, but future work will share results from longitudinal data and trends over the course of the 1-year study. All analyses were performed in SPSS Statistics v23 (IBM, Armonk, NY, USA). Descriptive statistics were performed on the remaining valid data. All data reported as mean (SD; range) unless indicated otherwise. All enrollment time data are in Coordinated Universal Time (UTC).

## Results

### Participant Enrollment

**Figure 2:**
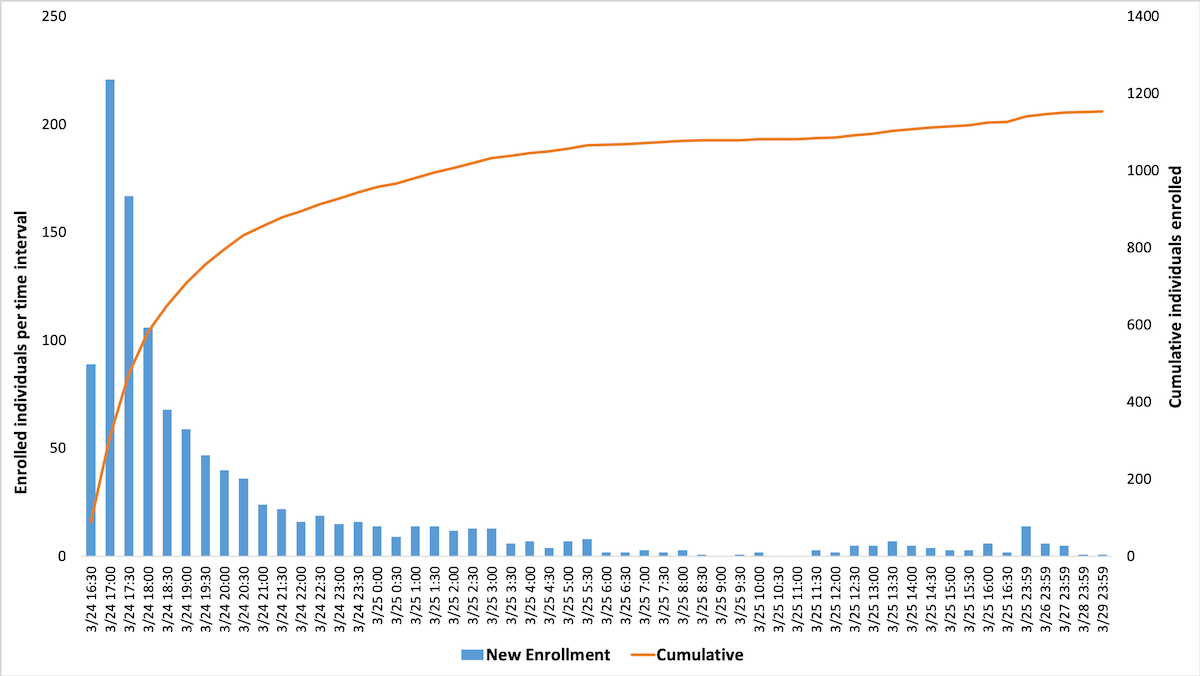
Participant enrollment rate by date and time interval.

Participants were first recruited by email around 9:00 am per Pacific Time Zone on March 24, 2017 (16:00 pm as per UTC). The study was closed to further enrollment on March 29, 2017. The first 100 participants were enrolled in less than 20 minutes. Enrollment reached 1000 participants within about 9 hours. Figure 2 provides a detailed bar graph of enrollment by date and time interval; blue bars represent the number of participants recruited at each time interval, and the red line indicates cumulative study enrollment. During the first 1 hour, the enrollment rate was 6.6 participants per minute (394 per hour). In the first 24 hours, the enrollment rate was 0.8 participants per minute (47 participants per hour).

**Figure 3:**
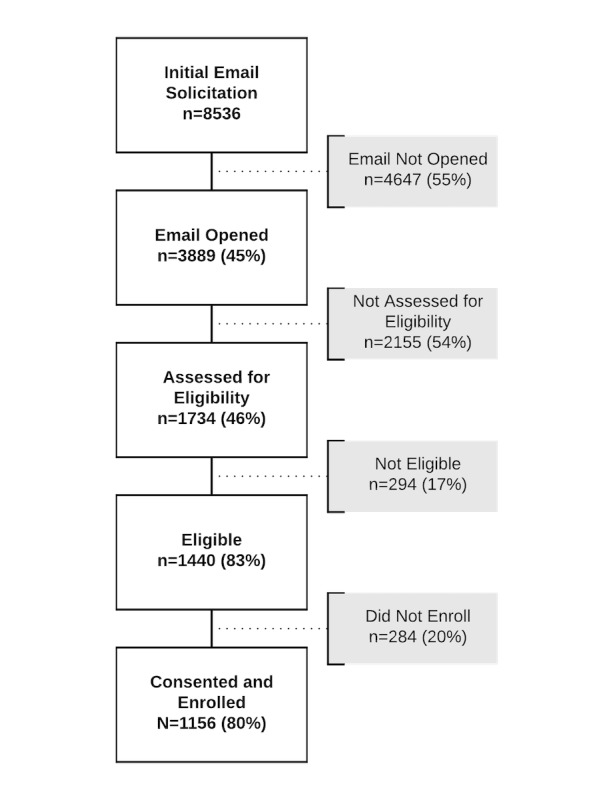
Study participant flow.

In total, 1156 participants were enrolled in the study within the recruitment window, but 1 withdrew shortly after enrollment, yielding 1155 participants. Complete study flow with the total number of participants at each step is documented in Figure 3. White boxes indicate steps of the enrollment process, with number and percentage of total valid participants; gray boxes indicate the numbers lost between each step.

**Figure 4:**
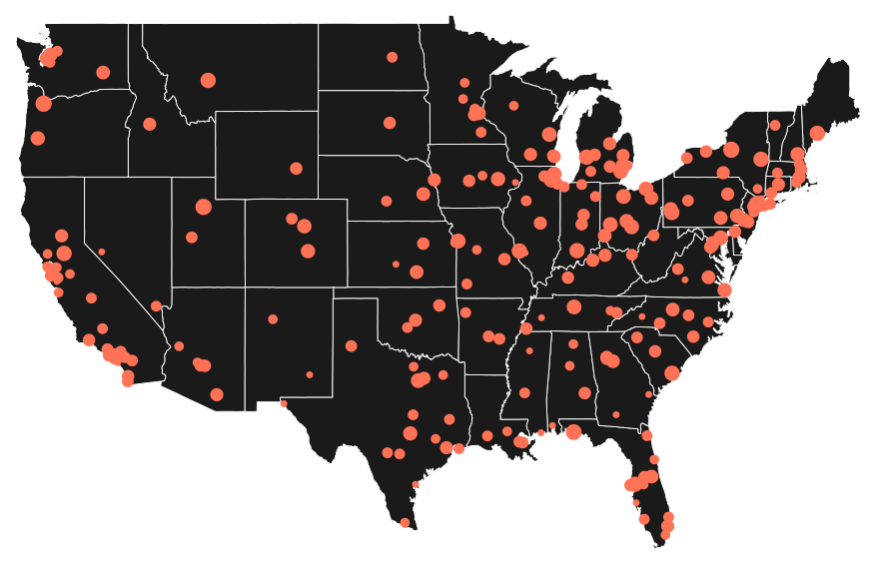
Geographic distribution of participants (point size scaled by the number of participants per area code).

In addition, the Web-based recruitment strategy attracted participants from a wide geographic distribution. Using telephone area codes as a proxy for location (which does not guarantee that participants live in these locations), at least 1 participant from all 50 states was enrolled in this study. A graphical representation of proxy geographic location is shown in Figure 4, with point size scaled by the number of participants per area code (range 1-12) and points located at the corresponding latitude and longitude centroid for each represented area code. Alaska and Hawaii area codes corresponded to 6 and 9 participants, respectively, but are not depicted. Furthermore, 12.0% (138/1153) of the participants had area codes from the 15 most rural states, defined by having >50% of the population residing in designated US Census Bureau rural settings [28].

### Questionnaire Engagement and Characteristics

Overall, 1132 participants completed the baseline questionnaires (1132/1156, 97.9%). Relevant participant demographic information is summarized in Table 1. Additional participant data are presented in Multimedia Appendix 1.

Mean participant age was 34.6 (9.4; 18-67) years. The mean participant weight was 183.4 (51.8; 94-425) pounds. The majority of the participants were women (1053/1132, 93.0%). Note that while Hispanic individuals were not specifically assessed in this study, participants had the opportunity to select “Other” and enter their race; 18 participants reported that they were Hispanic individuals.

### Sleepiness Checker Engagement

In this study, 1047 participants completed the baseline Sleepiness Checker activity (1047/1156, 90.5%). Mean days of the Sleepiness Checker completed per participant were 5.7 (1.7; 1-7). Overall, 51.8% (542/1047) of those who completed the baseline Sleepiness Checker completed it on all 7 days. A complete breakdown of the number of days that participants completed the Sleepiness Checker activity is outlined in Table 2.

### Objective Activity and Sleep Metrics

Overall, 1000 participants provided activity-specific Fitbit data (1000/1156, 86.5%), and 982 (982/1156, 84.9%) participants provided sleep-specific Fitbit data. Participants shared data from their Fitbits, and these data were used to provide objective sleep and activity metrics for the study.

**Table 1:** Sample characteristics.

Characteristic		Participants, n (%)		
		Total, n=1132	Females, n=1053	Males, n=79
**Education**				
	High school or general educational development	80 (7.1)	75 (7.1)	5 (6.3)
	Some college or 2-year degree	454 (40.1)	429 (40.7)	25 (31.6)
	4-year college degree	330 (29.2)	312 (29.6)	18 (22.8)
	More than 4-year college degree	265 (23.4)	238 (22.6)	26 (32.9)
**Household income in US $**				
	<10,000	47 (4.2)	44 (4.2)	3 (3.7)
	10,000-49,999	361 (31.9)	341 (32.4)	19 (24.1)
	50,000-99,999	406 (35.9)	384 (36.5)	22 (27.8)
	100,000-149,000	164 (14.5)	145 (13.8)	19 (24.1)
	150,000-199,999	45 (4.0)	39 (3.7)	6 (7.6)
	200,000-249,999	15 (1.3)	15 (1.4)	0 (0.0)
	250,000+	8 (0.7)	8 (0.8)	0 (0.0)
**Race**				
	White	979 (86.5)	923 (87.7)	56 (70.9)
	Black or African American	50 (4.4)	45 (4.3)	5 (6.3)
	Asian	36 (3.2)	28 (2.7)	8 (10.1)
	Other	29 (2.6)	25 (2.4)	4 (5.1)
	American Indian or Alaska Native	21 (1.9)	20 (1.9)	1 (1.3)
	Pacific Islander	9 (0.8)	5 (0.5)	4 (5.1)
**Marital status**				
	Married	570 (50.3)	531 (50.4)	39 (49.4)
	Never married	300 (26.5)	276 (26.2)	23 (29.1)
	Unmarried and living with partner	152 (13.4)	146 (13.9)	6 (7.6)
	Divorced	84 (7.4)	78 (7.4)	6 (7.6)
	Widowed	12 (1.1)	12 (1.1)	0 (0.0)
	Separated	11 (1.0)	11 (1.0)	0 (0.0)

**Table 2:** Participants’ Sleepiness Checker engagement (n=1047).

Day	Participants, n (%)
7	542 (51.8)
6	174 (16.6)
5	116 (11.1)
4	67 (6.4)
3	70 (6.7)
2	37 (3.5)
1	41 (3.9)

## Discussion

### Principal Results

This study resulted in several key findings in speeding up research enrollment while maintaining a high rate of engagement with the study protocol. The study enrolled 1156 participants within a 5-day window, with the majority being enrolled within the first day. Of the original 1156, only 1 withdrew from the study within the first week, indicating that the study was able to achieve a very low rate of initial dropout, which is important in conducting Web-based, eConsent studies. Finally, the rate of initial data collection was quite high for this type of research study as well, with 97.9% (1132/1156) of participants completing initial baseline questionnaires, 90.6% (1047/1156) completing the daytime Sleepiness Checker, and 84.9% (982/1156) sharing their wearable data.

### Comparison With Prior Work

Recruitment through the Achievement Studies platform yielded a remarkably high rate of enrollment, which can be attributed to (1) its Web-based methods and (2) its broad potential patient population. Compared with traditional cohort studies of sleep, which in line with previously introduced literature may take many months to enroll fully, Web-based methods of recruitment and enrollment can accelerate this process by orders of magnitude. The emerging Web-based study literature has many examples of this benefit in different patient populations. For instance, Web-based mental health studies with a similar number of participants reached targets in 5 months [29], 3 months [30], and 4 days [31]. A review of 12 studies utilizing Web-based recruitment methods calculated an average of 5 months spent on recruiting, ranging from 7 weeks to 7 months [32]. In comparison, our recruitment approach reached these targets within hours of the first email solicitation. Importantly, this study of sleep health had minimal inclusion and exclusion criteria and barriers to entry, as the study’s research focus is relevant to all individuals regardless of demographics or comorbidities. Such broad relevance, in addition to the convenient and predominantly passive means of data collection, additionally affects the observed recruitment timeframe.

It should be noted that one of the original ResearchKit studies (Health eHeart) reportedly enrolled over 10,000 new participants in its first 24 hours of study launch. However, several unique characteristics to its study launch were as follows: (1) the study team was able to work on the open-source platform for nearly a year before launch; (2) the launch was coordinated with the release of 4 other ResearchKit studies; and (3) it was announced by Apple at a major event.

While there are clear advantages to this method of participant recruitment and enrollment, some authors assert that significant drawbacks are involved in Web-based studies such as low levels of engagement and retention [32,33]. Long-term retention and engagement metrics for this study will be reported in subsequent publications; however, results demonstrate promisingly high levels of daily participant engagement, and other studies run on the Achievement Studies platform have yielded high completion rates [14,15]. Furthermore, Web-based recruitment methods are not subject to significant geographic restrictions and are limited only by access to the internet.

### Limitations

One limitation of this study is that it was conducted only with members of a Web-based community. While similar studies on the Achievement Studies platform can extend recruitment beyond just existing Achievement community members, to supplement with recruitment on other Web-based channels, on the occasion that the Achievement community is not deemed representative of the desired population, conducting research in this manner does require participants to have internet access. Additionally, the data sources required for this study limited participants to those who owned and used wearable devices. Another limitation is the sample’s limited demographic representativeness of the overall population. Participants were predominantly white women (largest subset married with children, educated, income >50k). Screening for the study was not implemented toward specific demographic targets; the inclusion criteria were those aged 18 years and above, residing in the United States, and able to read and understand English. A more diverse sample could have been quickly recruited through the Web by proportionally targeting subgroups using specific criteria, and this approach has been successfully used in other studies on the Achievement Studies platform. Furthermore, it is understood that objective data provided by consumer wearable devices may not accurately agree with clinically acquired measures, such as those obtained using actigraphy and polysomnography, although there is a growing body of recent literature pursuing validation efforts against gold standard, suggesting that consumer wearables may provide useful information about sleep and activity, especially with regards to intrauser trends [34,35]. Clearly, more research is required in the domain of consumer wearables [36-39]. Additional limitations involved in carrying out research in this manner are selection biases, the potential for low retention, and the possibility of privacy and security issues [40,41]. Moreover, participant geographical location was obtained through a proxy measure (telephone area code). This may not always reflect the geographic location where participants currently reside.

### Conclusions

The Achievement Studies platform is a useful framework for carrying out longitudinal research from which a large number of participants with wearable and other behavioral data can be rapidly recruited for research studies. In addition, initial participant retention and engagement appear promising. Carrying out community-based research in which individuals can be targeted for studies using specific criteria is an innovative approach to carrying out research. As is the case with large datasets, the data quality will need to be carefully examined. In addition, the representativeness of the sample of the overall population will need to be evaluated and long-term retention and engagement remains to be seen.

## Appendix

Multimedia Appendix 1 Supplemental data (n=1132).

## Abbreviations

eConsent: electronic informed consent
mHealth: mobile health
UTC: Coordinated Universal Time
