# Supplementary material for: Accelerating Research With Technology: Rapid Recruitment for a Large-Scale Web-Based Sleep Study
Source: JMIR Res Protoc. 2019 Jan 21;8(1):e10974. doi: 10.2196/10974 (PMC6360390; doi:10.2196/10974)
Supplement: Multimedia Appendix 1 [file resprot_v8i1e10974_app1.pdf]

Multimedia Appendix

Supplemental Data (n=1132).

**Main Daily Activities**	**Percent**
Work Full-time (day shifts)	58%
Stay at Home Parent	13%
Student	7%
Work Part-time (day shifts)	7%
Work Full-time (rotating/night shifts)	7%
Unemployed	3%
Work Part-Time (rotating/night shifts)	3%
Disabled	2%
Retired	1%
Work Schedule (Past 3 Months)	
Regular Day Shifts	61%
Irregular Shifts	19%
Rotating Shifts	5%
Regular Evening Shifts	4%
Split Shifts	2%
Regular Night Shifts	2%
Flexible Work Hours?	
No	52%
Yes	43%
Difficulty Paying for Basic Expenses	
Not Difficult	62%
Somewhat Difficult	27%
Difficult	5%
Very Difficult	3%
I Have a Good Life	
Agree	61%
Strongly Agree	23%
Neutral	11%
Strongly Disagree	4%
Disagree	2%
Weekly Alcohol Consumption	
None	51%
1-7 Drinks	44%
8-14 Drinks	3%

15-21 Drinks	2%
22-28 Drinks	2%
>28 Drinks	1%
Smoking Status	
Never Smoked	71%
Former Smoker	19%
Daily Smoker	4%
Occasional Smoker	2%
Light Smoker	1%
Smoking Frequency	
Never	92%
Every Day	5%
Some Days	3%
General Health	
Good	43%
Very Good	37%
Fair	12%
Excellent	7%
Poor	2%
Change In Health In A Year	
Same As A Year Ago	46%
Somewhat Better	28%
Much Better	13%
Somewhat Worse	12%
Much Worse	1%
Medical Conditions	
Allergies	54%
Depression	40%
Anxiety	37%
Asthma	23%
Insomnia	20%
Obstructive Sleep Apnea	6%
